# Bcl11 Transcription Factors Regulate Cortical Development and Function

**DOI:** 10.3389/fnmol.2020.00051

**Published:** 2020-04-08

**Authors:** Ruth Simon, Christoph Wiegreffe, Stefan Britsch

**Affiliations:** Institute of Molecular and Cellular Anatomy, Ulm University, Germany

**Keywords:** Bcl11a, Bcl11b, transcription factors, brain development, hippocampus, neocortex, neural circuitry, neurological disorders

## Abstract

Transcription factors regulate multiple processes during brain development and in the adult brain, from brain patterning to differentiation and maturation of highly specialized neurons as well as establishing and maintaining the functional neuronal connectivity. The members of the zinc-finger transcription factor family Bcl11 are mainly expressed in the hematopoietic and central nervous systems regulating the expression of numerous genes involved in a wide range of pathways. In the brain Bcl11 proteins are required to regulate progenitor cell proliferation as well as differentiation, migration, and functional integration of neural cells. Mutations of the human Bcl11 genes lead to anomalies in multiple systems including neurodevelopmental impairments like intellectual disabilities and autism spectrum disorders.

## Transcription Factors Regulating Brain Development and Function

Transcription factors (TFs) play a crucial role during development as well as in the adult organism. Early on during embryogenesis the precisely regulated temporal and spatial expression of TFs is required to establish the body plan laying the foundation for the different tissues as well as regulating physiological functions. The fine-tuned expression of TFs is of particular importance during development and maintenance of the brain (Nord et al., 2015). TFs not only are necessary to define the specificity of neurons but also are indispensable for generating the correct wiring among the different neuronal cells allowing the highly specialized connectivity and plasticity of the brain (Hsieh, 2012; Urban and Guillemot, 2014). Deregulation of TF expression results in a broad range of diseases including neurodevelopmental diseases like autism spectrum disorder (De Rubeis et al., 2014) and neurodegenerative diseases like Alzheimer’s, Schizophrenia and Huntington’s disease (Desplats et al., 2008; Dard et al., 2018; Whitton et al., 2018).

Here we focus on the zinc-finger transcription factor family Bcl11 expressed mainly in the hematopoietic and the central nervous systems. The crucial role of the Bcl11 genes in brain development and function became apparent by recent reports describing patients carrying heterozygous mutations of Bcl11 and their implications in intellectual disabilities and neurological disorders (Dias et al., 2016; Punwani et al., 2016; Lessel et al., 2018). Emerging evidence demonstrates that Bcl11 proteins execute important functions during central nervous system development and in adult neurogenesis (Arlotta et al., 2005, 2008; Kuo et al., 2009, 2010a,b; John et al., 2012; Simon et al., 2012, 2016; Canovas et al., 2015; Wiegreffe et al., 2015; Greig et al., 2016; Woodworth et al., 2016; De Bruyckere et al., 2018). However, the molecular mechanisms required to establish and maintain these functional connections are not yet fully understood. This review provides an overview of Bcl11 regulation of molecular mechanisms in the brain, focusing in particular on Bcl11 functions in different cortical regions, i.e., hippocampus and neocortex. A better understanding of these mechanisms could contribute to the development of therapeutic treatments to prevent and/or cure neurological disorders as proposed in recent reports (Choi et al., 2018; Dard et al., 2018).

## The Bcl11 Zinc-Finger Transcription Factor Family

**FIGURE 1:**
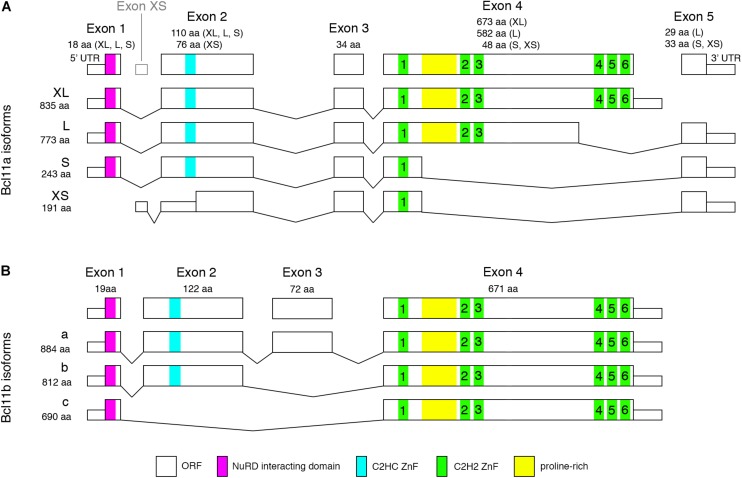
Mouse Bcl11 predicted protein isoforms. **(A)** Alternative splicing of Bcl11a leads to four isoforms containing 1, 3, or 6 C2H2 zinc-finger domains required for DNA-binding. Exon 1 and 2 are common to all but the Bcl11a-XS isoform containing the NuRD interacting domain and a C2HC zinc-finger domain involved in protein-protein interaction. **(B)** Alternative splicing of Bcl11b results in three isoforms containing either all four exons or lacking exon 3 or exon 2 and 3. All Bcl11b isoforms contain the C2H2 zinc-finger domains and NuRD interacting domain. Bcl11b isoform a and b retain the C2HC zinc-finger domain which is missing in isoform c.

The Bcl11 genes were first identified by their functions in the immune system, Bcl11a also known as Ctip1 as a proto-oncogene and Bcl11b or Ctip2 as a tumor suppressor gene (Avram et al., 2000; Satterwhite et al., 2001). The Bcl11 genes are Krüppel-like sequence-specific C2H2 zinc-finger transcription factors located on chromosome 11 and 12 in the mouse and 2 and 14 in humans, respectively. Bcl11 genes are highly conserved throughout evolution as was shown for the human Bcl11a gene sharing 95% homology with the mouse, chicken, and Xenopus genes and 67 and 61% homology on nucleotide and protein levels with Bcl11b, respectively (Satterwhite et al., 2001). In addition to the C2H2 zinc-finger domains located on exon 4 of both genes, Bcl11 proteins contain several protein-protein interacting domains at their N-terminal end like the nucleosome remodeling and deacetylase (NuRD) interacting domain and a CCHC finger motif (Figure 1; Liu et al., 2006). The CCHC motif unlike the DNA-binding C2H2 motif promotes dimerization and nuclear translocation allowing the transcriptional regulation of target genes as was shown for Bcl11b (Grabarczyk et al., 2018). Furthermore, analysis of heterozygous mutations of the human CCHC finger motif strongly suggests that Bcl11b functions as a dimer (Grabarczyk et al., 2018). Although no data are available so far it is most likely that the CCHC motif has a similar function for Bcl11a protein regulation.

Alternative splicing of Bcl11 mRNAs results in at least four isoforms of human and mouse Bcl11a as well as 2 and 3 isoforms of Bcl11b in human and mouse, respectively (Figure 1). The resulting Bcl11a proteins contain none (human Bcl11a-XS, 142aa), one (mouse Bcl11a-XS, 191aa; human and mouse Bcl11a-S, 243aa), three (human and mouse Bcl11a-L, 773aa) or all six (human and mouse Bcl11a-XL, 835aa) C2H2 zinc finger domains (Figure 1A; Satterwhite et al., 2001; Liu et al., 2006). All isoforms contain the NuRD interacting and CCHC zinc-finger domains except the mouse Bc11a-XS isoform lacking both motifs (Figure 1A). The human Bcl11b presents two isoforms (894aa and 823aa) containing or lacking exon 3 (Lennon et al., 2016) while the mouse Bcl11b isoforms are containing all four exons (isoform a, 884aa) or lacking either exon 3 (isoform b, 812aa) or exon 2 and 3 (isoform c, 690aa) (Figure 1B). All human and mouse Bcl11b/Ctip2 isoforms retain the six C2H2 zinc-finger domains as well as the NuRD interacting and CCHC domains with the exception of mouse isoform c lacking the CCHC motif (Figure 1B). The isoforms are expressed to various degrees in different tissues but so far, no specific function is assigned to individual isoforms in the brain. Possible functions could include self-regulation of Bcl11 protein activity by assembling of diverse dimers and protein complexes comprised of specific isoforms or factors like the NuRD complex allowing or preventing nuclear translocation and/or DNA binding. Interestingly, early work by Nakamura et al. showed that short isoforms of Bcl11a are restricted to cytoplasm whereas longer isoforms are located in the nucleus (Nakamura et al., 2000) thus being compatible with a possible modulating function of the transcriptional activity of a Bcl11 protein complex.

**FIGURE 2:**
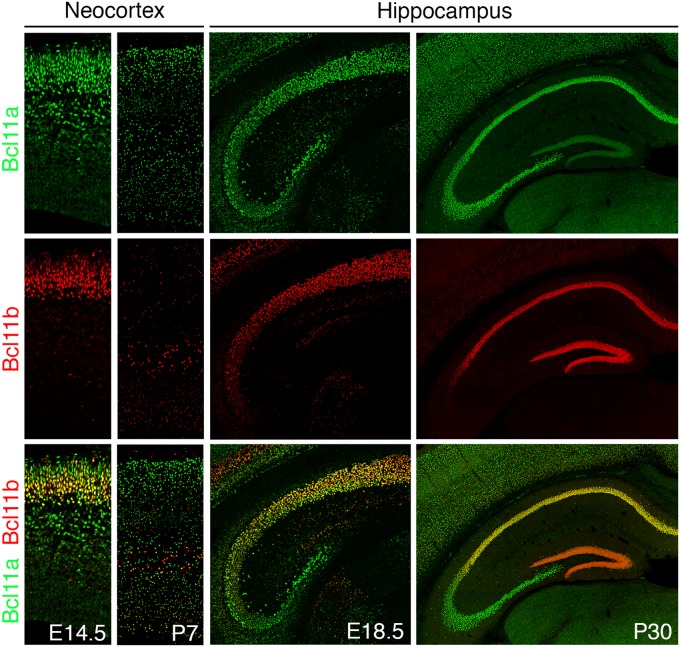
Bcl11 expression in the mouse brain. Bcl11 gene expression occurs early in development continuing to adulthood as shown here for the neocortex (left panels) and the hippocampus (right panels) by immuno-histochemical staining. Bcl11a (green) and Bcl11b (red) expression are shown in the neocortex at embryonic stage 14.5 (E14.5) and postnatal stage 7 (P7) and in the hippocampus at embryonic stage 18.5 (E18.5) and postnatal stage 30 (P30).

Both Bcl11 genes are expressed during embryonic development as early as E10.5 and continuing to be expressed throughout life in several tissues like the brain, immune system, and skin (Leid et al., 2004; Golonzhka et al., 2007; Zhang et al., 2012). In the central nervous system, Bcl11 genes are expressed in the dorsal spinal cord, neocortex, hippocampus, entorhinal cortex, striatum, amygdala, and cerebellum (Table 1; Leid et al., 2004; Arlotta et al., 2005, 2008; Desplats et al., 2008; John et al., 2012; Simon et al., 2012; Canovas et al., 2015; Surmeli et al., 2015; Wiegreffe et al., 2015; Greig et al., 2016; Woodworth et al., 2016; Ohara et al., 2018). The best studied brain areas in relation to Bcl11 are the hippocampus, neocortex, spinal cord, and striatum. In the striatum Bcl11b has an important function in the differentiation of medium spiny neurons which have a critical role in motor control (Arlotta et al., 2008). In other brain areas like the cerebellum and entorhinal cortex both Bcl11 genes are expressed but so far, no distinct functions were determined. Expression analyses revealed a partially overlapping but also distinct expression pattern of both genes in the brain (Figure 2). For example, during early corticogenesis Bcl11a and Bcl11b are co-expressed by the majority of post-mitotic neurons in the intermediate zone and cortical plate. However, already at late embryonic stages two developmental lineages emerge with mutually exclusive expression of either transcription factor, which persists into adulthood (Figure 2, left panels; Woodworth et al., 2016). In the hippocampus on the other hand the expression pattern is established early on with an overlapping expression of both Bcl11 genes in CA1/2 regions and distinct expression of Bcl11a in CA3 and Bcl11b in the dentate gyrus, respectively (Figure 2, right panels; Leid et al., 2004; Simon et al., 2012).

**TABLE 1:** Expression of Bcl11a and Bcl11b in regions of the central nervous system.

CNS region	Expression		Phenotype		References
		
	Bcl11a	Bcl11b	Bcl11a	Bcl11b	
Neocortex	+	+	+	+	Leid et al., 2004; Arlotta et al., 2005; Canovas et al., 2015; Wiegreffe et al., 2015; Woodworth et al., 2016; Greig et al., 2016
Olfactory cortex	+	+	n.d.	n.d.	Leid et al., 2004
CA1-2	+	+	n.d.	-	Leid et al., 2004; Simon et al., 2012
CA3	+	-	n.d.	-	Leid et al., 2004; Simon et al., 2012; Chan et al., 2013
Dentate gyrus	-	+	-	+	Leid et al., 2004; Simon et al., 2012, 2016; De Bruyckere et al., 2018
Entorhinal cortex	+	+	n.d.	n.d.	Surmeli et al., 2015; Ohara et al., 2018
Striatum	+	+	n.d.	+	Arlotta et al., 2008; Desplats et al., 2008
Amygdala	-	+	n.d.	n.d.	Leid et al., 2004
Thalamus	+	-	n.d.	n.d.	Leid et al., 2004
Hypothalamus	+	-	n.d.	n.d.	Leid et al., 2004
Inferior colliculus	-	+	n.d.	n.d.	Leid et al., 2004
Cerebellum	+	+	n.d.	n.d.	Leid et al., 2004
Pons	+	-	n.d.	n.d.	Leid et al., 2004
Spinal cord	+	+	+	n.d.	Leid et al., 2004; John et al., 2012

*n.d., not determined.*

Little is known about the upstream control of Bcl11 expression in the brain. Best studied is the regulation of Bcl11b expression by Satb2, a chromatin regulating protein binding to the matrix attached region (MAR) of the Bcl11b locus (Leone et al., 2015). Satb2 builds a multi-protein complex with other chromatin regulating proteins like the NuRD complex modulating the Bcl11b locus and repressing transcription in a time- and region-specific manner (Chen et al., 2008; Baranek et al., 2012; McKenna et al., 2015; Leone et al., 2015; Harb et al., 2016). The transcription factor Tbr1, expressed in excitatory neurons, also is involved in the regulation of Bcl11 expression during neocortex development. Yet, it remains to be determined whether Tbr1 directly regulates the Bcl11 genes (Hevner et al., 2001; Sanders et al., 2015; Fazel Darbandi et al., 2018). In contrast to the nervous system, upstream control of Bcl11 proteins has been more extensively studied in the lympho-hematopoietic system (for comprehensive review see Bauer and Orkin, 2015). In erythroid cells, for example, developmental expression of Bcl11a is regulated by the RNA-binding protein Lin28b (Basak et al., 2020). Whether similar regulatory mechanisms control the translation of Bcl11 proteins in neurons has not been investigated.

## Bcl11 Regulation of Target Genes

The transcriptional regulation of target genes by Bcl11 proteins requires binding of the C-terminal zinc-finger domains to specific DNA binding motifs. Bcl11a recognizes the GGCCGGAGG motif and Bcl11b GGCCG/AG/AGG, a variation of this motif, repressing the expression of target genes independently of histone deacetylases (Avram et al., 2002). However, Bcl11 transcription factors not only act as transcriptional repressors but also as activators. This was shown for Bcl11b binding in a complex with p300 to IL-2 and Cot kinase genes in CD4^+^ T-cells recognizing TGGGC as DNA binding motif (Cismasiu et al., 2006, 2009). From these data one could assume that promoters of Bcl11 target genes containing the GGCCGGAGG motif are transcriptionally inhibited and promoters containing the TGGGC motif are activated by Bcl11 proteins. This was refuted by the presence of both motifs in promoter sequences of activated or repressed target genes recognized by both Bcl11 proteins (John et al., 2012; Simon et al., 2012; Wiegreffe et al., 2015; De Bruyckere et al., 2018). In addition, the binding of Bcl11 proteins was more efficient when both motifs were present as was shown for desmoplakin (Dsp), a direct Bcl11b target gene activated during dentate gyrus development (Simon et al., 2012), and Sema3c, directly regulated by Bcl11a (Wiegreffe et al., 2015). In the case of Sema3c the promoter sequence consists of six repeats of the combined GGCCGG and TGGGC motifs, TGGGCCGG, embedded in a 212 base pair repeat sequence (Wiegreffe et al., 2015). More recently it was shown by an unbiased *in vitro* approach that both, Bcl11a and Bcl11b zinc-finger domains bind with high affinity to a TGACCA motif (Liu N. et al., 2018). The different regulatory mechanisms of Bcl11 proteins suggest that the transcriptional regulation requires a binding complex consisting of Bcl11 and additional specific co-factors to either activate or inhibit the expression of particular target genes.

## Bcl11 Binding Complex

Both Bcl11 proteins were independently isolated through their interaction with the chicken ovalbumin upstream promoter transcription factor (COUP-TF) of orphan nuclear receptors (Avram et al., 2000, 2002; Senawong et al., 2003). Both proteins repress the expression of their target genes when in a complex with COUP-TF or the NuRD complex (Cismasiu et al., 2005). NuRD complex is a major ATP-dependent chromatin remodeling complex consisting of a range of subunits like the metastasis-associated genes (MTAs) allowing the transcriptional regulation of target genes by binding tissue specific transcription factors (Lai and Wade, 2011; Kumar and Wang, 2016). This was shown for the binding of the Bcl11b-NuRD complex to its target gene p57KIP2, a cyclin dependent kinase, involving co-binding of MTA2 and HDAC2 (Topark-Ngarm et al., 2006). The repressor function of Bcl11b is executed by binding to the MTAs via the conserved N-terminal MSRRKQ motif (Cismasiu et al., 2005; Dubuissez et al., 2016). Phosphorylation of the serine of the MSRRKQ motif impedes Bcl11b binding to MTAs preventing transcriptional repression of target genes (Dubuissez et al., 2016). Furthermore, both Bcl11 proteins mediate transcriptional repression *in vitro* through recruitment of the histone deacetylase SIRT1 (Senawong et al., 2003, 2005). Moreover, Bcl11a interacts with members of the NR2E/F subfamily of nuclear receptors, through two distinct regions containing a novel signature motif (F/YSXXLXXL/Y) (Chan et al., 2013). Using a proteomics approach several Bcl11a-interacting partners were identified in erythroid cells, including components of the NuRD, LSD1/CoREST, and SWI/SNF or BAF complexes (Xu and Henkemeyer, 2009).

Up to now the described complex comprised of Bcl11 proteins and chromatin remodeling factors were studied mostly in the hematopoietic system which does not exclude a role of these factors in the transcriptional regulation of the nervous system. However, during neuronal development the SWI/SNF or BAF complex consisting of several subunits including the Bcl11 proteins (BAF100a/b), seems to play a more crucial role. First discovered as tumor suppressor genes involved in a range of cancers (Kadoch et al., 2013; Kadoch and Crabtree, 2015) some of the subunits carry out distinct functions during neuronal development and their dysfunction leads to neurodevelopmental disorders including intellectual disability (Bogershausen and Wollnik, 2018). Different compositions of the BAF complex are present at different embryonic as well as neuronal differentiation and maturation stages (Son and Crabtree, 2014; Sokpor et al., 2017). Regarding the role of Bcl11b in dentate gyrus development and adult neurogenesis two neuron-specific BAF family members, BAF53b and BAF170, are of specific interest. BAF170 is expressed in neuronal progenitor cells playing a role in the regulation of neurogenesis in the developing as well as the adult dentate gyrus. Loss of BAF170 expression in the hippocampus affects the pool of progenitor cells and neuronal differentiation resulting in impaired learning behavior (Tuoc et al., 2017). BAF53b on the other hand is expressed in post-mitotic neurons and involved in synaptic plasticity of the hippocampus and long-term memory consolidation (Vogel-Ciernia et al., 2013; Vogel-Ciernia and Wood, 2014; Yoo et al., 2017). The phenotype analysis of these BAF members shows similarities to the hippocampal Bcl11b phenotype (Simon et al., 2012, 2016; De Bruyckere et al., 2018). It would be of interest to directly determine how these BAF family members and Bcl11b interact during postnatal dentate gyrus development and adult neurogenesis.

## Bcl11a Regulation of Spinal Cord and Neocortex Development

Conditional mutagenesis of Bcl11a in the developing dorsal spinal cord of the mouse demonstrated impaired morphological differentiation interfering with the formation of the somatosensory circuitry within the dorsal spinal horn. This connectivity is in part established by the Wnt pathway component Frzb/Sfrp3, which was identified as the first functional downstream target of Bcl11a during CNS development (John et al., 2012). Bcl11a is also expressed by young post-mitotic projection neurons of the developing neocortex as early as E12.5 (Figure 2, left panels). Projection neurons are born in germinal zones located near the ventricles and undergo radial migration toward the pial surface of the brain changing from a multipolar to a bipolar cell shape on the way (Noctor et al., 2004). Late-born Bcl11a deficient neurons destined to settle in superficial layers fail to switch from multipolar to bipolar morphology and undergo delayed migration into the cortical plate (Wiegreffe et al., 2015). Bcl11a directly represses Semaphorin 3c (Sema3c) expression, which is upregulated in the intermediate zone of the Bcl11a deficient neocortex. Normalization of Sema3c expression in Bcl11a deficient projection neurons restores radial migration in these cells. Moreover, Bcl11a binds to a DNA repeat in the second intron of Sema3c that conveys transcriptional repression. Thus, Bcl11a acts through Sema3c as an important regulator of radial migration in developing cortical projection neurons (Wiegreffe et al., 2015).

Although Bcl11a deficient superficial neurons appear to be correctly specified at birth, changes were observed in the populations of projection neuron subtypes within Bcl11a deficient deep cortical layers. Here, supernumerary subcerebral neurons are born at the expense of callosal and corticothalamic neurons (Wiegreffe et al., 2015; Woodworth et al., 2016). In contrast, overexpression of Bcl11a in deep cortical layers suppresses subcerebral neuron identity and projection toward the spinal cord. Thus, Bcl11a appears to specify subtype identity in deep-layer cortical neurons, preventing corticothalamic and callosal projection neurons to acquire a subcerebral projection neuron identity (Woodworth et al., 2016). Contradicting this model, it was shown that reducing the expression levels of Bcl11a in deep cortical layers by small hairpin RNA leads to increased Tbr1 expression and decreased subcerebral neuron fate (Canovas et al., 2015). This discrepancy could be explained by residual expression of Bcl11a after knockdown as compared to genetic loss of function and highlights the general importance of balanced gene dosage of transcription factors for specification of neuron subtypes. As neocortical development proceeds, Bcl11a becomes refined and highly expressed in primary sensory areas across cortical layers VI, IV, and deeper II/III in wildtype brains (Greig et al., 2016). By using a transcriptomic approach, Greig et al. (2016) showed that area-specific genes, which are normally enriched in wildtype sensory cortex, are downregulated, whereas genes typical for the motor cortex are upregulated in Bcl11a deficient sensory cortex. This in turn leads to disturbances of cortical connectivity, including cortico-cortical and corticofugal axonal projections (Greig et al., 2016). In summary, Bcl11a plays an important role for various aspects of cortical development, including specification of early-born deep-layer projections neurons, radial migration and postnatal morphological differentiation of late-born superficial-layer projection neurons, and acquisition of cortical sensory area identity. Still, our knowledge about the specific downstream genetic programs, which are transcriptionally regulated by Bcl11a, is incomplete and awaits further investigation.

## Bcl11b and Hippocampal Development

The hippocampus consisting of the dentate gyrus (DG), the Cornu ammonis (CA), and the subiculum has an important function in learning and memory as well as emotional behavior (Garthe et al., 2009, 2016; Vivar et al., 2013). The DG, functioning as the primary gateway for input information, develops mainly postnatally under the control of stage-specific transcription factors (Hsieh, 2012; Urban and Guillemot, 2014; Berg et al., 2019). Hippocampal Bcl11b expression is first detected at E15, in the cornu ammonis and few cells of the developing dentate gyrus anlage, expanding to the suprapyramidal blade by E18. During postnatal development and throughout life Bcl11b is expressed in post-mitotic cells of the dentate gyrus as well as the pyramidal cells of the CA1 and CA2 regions but not the CA3 region (Figure 2, right panels; Simon et al., 2012). In the DG Bcl11b regulates progenitor cell proliferation, differentiation and maturation of neurons as well as their functional integration (Simon et al., 2012, 2016; De Bruyckere et al., 2018). Although Bcl11b is selectively expressed in post-mitotic granule cells, loss of Bcl11b expression affects progenitor cell proliferation suggesting a non-cell autonomous regulatory mechanism. In addition, deletion of Bcl11b causes an arrest of granule cell differentiation at the mitotic to post-mitotic transition stage implying a cell autonomous regulation of cell differentiation. Unexpectedly, Bcl11b activates the transcription of desmoplakin, a cell adhesion molecule, in dentate gyrus granule cells (Simon et al., 2012). Comparing the Bcl11b and desmoplakin phenotype suggests desmoplakin to be responsible for cell-cell communication to regulate proliferation and differentiation as well as spine formation (Simon et al., 2012).

Ablation of Bcl11b during postnatal development causes a dramatic mossy fiber phenotype implying a role in axon outgrowth and pruning (Simon et al., 2012). Mossy fibers, the axons of dentate gyrus granule cells, are required to relay information from the DG to the CA3. During development a number of axon guidance molecules including ephrins and semaphorins are involved to ensure a functional DG-CA3 circuit by directing the outgrowth as well as the pruning of mossy fibers (Bagri et al., 2003; Riccomagno et al., 2012; Liu X. D. et al., 2018). Bcl11b directly represses Sema5b expression which was shown to be involved in the elimination of excess mossy fiber synapses (O’Connor et al., 2009; De Bruyckere et al., 2018). Although Bcl11b is not expressed in CA3 the loss of Bcl11b expression in the dentate gyrus causes fewer thorny excrescences, the postsynaptic partner of mossy fiber boutons of CA3 pyramidal neurons (Simon et al., 2012).

## Bcl11b and Adult Hippocampal Neurogenesis

Since Altman and colleagues first reported about adult neurogenesis in the 1960s (Altman and Das, 1965) its existence was often questioned. While over the years strong evidence accumulated of adult neurogenesis occurring in rodents it has been more challenging to unambiguously demonstrate its existence in the human brain. A recent report declaring the stop of neurogenesis in childhood sparked again the discussion about adult-born neurons in humans (Sorrells et al., 2018). However, adjusting the lifespan of the different species and methods used lead to the conclusion that adult neurogenesis occurs in all species but drops to very low rates during postnatal life (Spalding et al., 2013; Kuipers et al., 2015; Boldrini et al., 2018; Kempermann et al., 2018; Moreno-Jimenez et al., 2019; Snyder, 2019). Numerous publications examining adult neurogenesis in rodents demonstrate an important function for newborn neurons in the adult or aged brain in regard to learning and memory as well as stress behavior (Garthe et al., 2009, 2016; Vivar et al., 2013; Jain et al., 2019).

The Bcl11b adult-induced mouse model provides further evidence for the importance of adult neurogenesis (Simon et al., 2016). Adult-induced Bcl11b mutants exhibit similarities to the postnatal phenotype with the exception of the mossy fiber tract which appeared not to be affected (Simon et al., 2012, 2016). However, a closer examination of the DG-CA3 connectivity revealed a reduced number of synapses and ultra-structural changes of the adult-induced Bcl11b mutant boutons which are mirrored by a dramatic decline of long-term potentiation as early as 2 weeks after induction of the mutation weeks before the onset of apoptosis and arrest of differentiation (De Bruyckere et al., 2018). These data demonstrated for the first time that transcriptional mechanisms directly regulate synaptic homeostasis independent of activity.

Given the Bcl11b mutant phenotype in mice, specifically changes of the dentate gyrus-CA3 connectivity, strongly suggests behavioral consequences. Indeed, analyzing hippocampal specific behavior of Bcl11b mouse models demonstrated impairment of spatial learning and working memory (Simon et al., 2012, 2016). These behavioral changes are more pronounced in the conditional than the adult-induced Bcl11b mutant most likely due to the more severe phenotype of the DG-CA3 circuitry. It seems that once established the mossy fiber tract is more resistant to interferences, e.g., loss of Bcl11b expression, in contrast to the developing mossy fiber tract. In addition, Bcl11b might execute different functions during postnatal development of the dentate gyrus and in the adult hippocampus. It seems that Bcl11b no longer plays such a crucial role in the regulation of progenitor cell proliferation in the adult hippocampus but is required for the maintenance of the existing and integrated neurons (Simon et al., 2016). Although the DG-CA3 circuitry is affected in adult-induced Bcl11b mutants it appears that the remaining dentate gyrus granule cells are able to compensate for the loss preventing a more striking behavioral phenotype.

## Bcl11 Transcription Factors and Neurological Disorders

Recent reports examining patients carrying heterozygous Bcl11 mutations not only revealed severe defects of the immune system but also neurodevelopmental disorders including intellectual disabilities (Dias et al., 2016; Punwani et al., 2016; Lessel et al., 2018; Soblet et al., 2018; Yoshida et al., 2018; Peron et al., 2019). In the case of Bcl11a the mutations analyzed so far reside at the amino terminal end most likely preventing protein-protein interactions, e.g., homodimerization for nuclear localization and complex building of factors required for DNA binding, leading to impaired transcriptional regulation (Dias et al., 2016). In addition, the 2p15-16.1 microdeletion syndrome affecting also the Bcl11a gene leads to brain abnormalities like hypoplasia of the corpus callosum, neocortex, amygdala as well as hippocampus (Rajcan-Separovic et al., 2007; Hancarova et al., 2013; Peter et al., 2014; Balci et al., 2015; Bagheri et al., 2016). Moreover, Bcl11a was reported by several large-scale exome sequencing studies to be a candidate risk gene for neuropsychiatric disorders, including intellectual disability and autism spectrum disorders (ASDs) that are linked with impaired neocortical development (Cooper et al., 2011; Iossifov et al., 2012; De Rubeis et al., 2014; Schizophrenia Working Group of the Psychiatric Genomics Consortium, 2014; Deciphering Developmental and Disorders Study, 2015). Reports concerning Bcl11b described frameshift, nonsense and missense mutations as well as chromosomal rearrangements located in the center part or the C-terminal end of the protein most likely affecting the DNA binding capacity (Punwani et al., 2016; Lessel et al., 2018). These mutations resulted in multisystem anomalies, immune deficiency, developmental delay as well as intellectual disabilities with varying degrees of severity probably depending on the mutation site (Punwani et al., 2016; Lessel et al., 2018).

Notably, autism spectrum disorders could also be caused by impaired remodeling of the BAF complex (Ronan et al., 2013; Vogel-Ciernia and Wood, 2014; Sokpor et al., 2017; Alfert et al., 2019). Bcl11 proteins as part of the BAF complex are associated with autism spectrum disorder and schizophrenia (Basak et al., 2015; Dias et al., 2016; Sokpor et al., 2017). During neuronal development specific BAF complexes are assembled at different stages: embryonic stem cell (esBAF), progenitor (pnBAF), and neuronal BAF (nBAF) complexes. These complexes differ by the composition and ratio of specific subunits. As an example, BAF53a in pnBAF is replaced by BAF53b in nBAF complexes which coincides with the transition from mitotic to post-mitotic stages of the cell (Alfert et al., 2019). Failure of this replacement of factors results in impaired progenitor cell proliferation and dendritic morphogenesis as well as learning and memory impairment (Vogel-Ciernia et al., 2013; Vogel Ciernia et al., 2017).

Examining the adult and aging human as well as rodent brain suggests Bcl11b to be involved in a number of neurodegenerative disorders like Alzheimer’s disease (AD) (Desplats et al., 2008; Choi et al., 2018; Dard et al., 2018; Llorens-Martin, 2018), Huntington’s disease (HD) (Desplats et al., 2008; Ahmed et al., 2015), schizophrenia (Whitton et al., 2016, 2018), and amyotrophic lateral sclerosis (ALS) (Chesi et al., 2013; Lennon et al., 2016). One common feature of neurodegenerative diseases including HD, AD, Parkinson disease as well as ALS, is the loss of synapses (Hong et al., 2016; Bae and Kim, 2017; Sellgren et al., 2019). Synapses are the most crucial structure for neuronal communication and loss of synapses, in particular alterations of the presynaptic terminal are highly indicative of neural diseases (Bae and Kim, 2017). In addition, deregulated synapse elimination or pruning during development leads to neurodevelopmental diseases like ASD and schizophrenia (Paolicelli et al., 2011; Stephan et al., 2012). Bcl11b is required for the formation and maintenance of synapses during development as well as in adulthood in the hippocampus possibly involving a member of the C1ql gene family, C1ql2 (Simon et al., 2012; De Bruyckere et al., 2018). C1q-like proteins (C1ql1-4), a subgroup of the C1q gene family, are involved in the formation and stabilization of synapses acting as extracellular scaffolding proteins and regulating synaptic activity by establishing among others postsynaptic kainate-type glutamate receptor complexes (Matsuda et al., 2016; Matsuda, 2017; Yuzaki, 2017, 2018). Elimination of non-functional synapses on the other hand is mediated by microglia and other members of C1q family proteins (Stephan et al., 2012; Hong et al., 2016; Henstridge et al., 2019; Lee and Chung, 2019; Sellgren et al., 2019; Wilton et al., 2019). Loss of Bcl11b expression might contribute to the onset of neurological disorders by direct prevention of synapse formation and maintenance and indirect activation of synapse elimination by microglia and C1q proteins.

## Concluding Remarks

In the nervous system Bcl11 proteins regulate multitudes of signaling pathways during development, maturation and aging establishing and maintaining a functional brain circuitry. Both Bcl11 proteins are expressed in a number of diverse cells regulating their proliferation, differentiation and maturation by conserved mechanisms. However, numerous unanswered questions remain concerning the molecular mechanisms of Bcl11 regulation. How do the widely expressed Bcl11 genes direct the development and maintenance of highly specialized cells like projection neurons in the brain? Most importantly, what are the partners of Bcl11 in these processes, e.g., downstream genes and co-factors of specific binding complexes to ensure the specific fate of cells? Answers could be provided by comparative transcriptome analyses of single cells determining target genes and signaling pathways as well as proteomics to determine the composition of specific protein complexes involved. Interestingly, human Bcl11 mutations result in neurodevelopmental impairments including autism spectrum disorder. Reiterating human Bcl11 mutations in mouse models could further contribute to a better understanding of the involved molecular mechanisms. Lessel et al. (2018) introduced genetically human-type Bcl11b mutations into the mouse and compared the phenotypic consequences in both species. They could demonstrate that human and mouse Bcl11b mutations have comparable regulatory functions in mouse brain development (Lessel et al., 2018). Thus, the mouse model provides a promising approach to determine in depth Bcl11a/b dependent regulatory pathways in the brain.

## Author Contributions

RS wrote main part of the manuscript. CW contributed to the text and made figures. SB contributed to the text.

## Conflict of Interest

The authors declare that the research was conducted in the absence of any commercial or financial relationships that could be construed as a potential conflict of interest.

## References

[B1] Ahmed I. Sbodio J. I. Harraz M. M. Tyagi R. Grima J. C. Albacarys L. K. (2015). Huntington’s disease: neural dysfunction linked to inositol polyphosphate multikinase. *Proc. Natl. Acad. Sci. U.S.A.* 112 9751–9756. 10.1073/pnas.1511810112 26195796 PMC4534278

[B2] Alfert A. Moreno N. Kerl K. (2019). The BAF complex in development and disease. *Epigenetics Chromatin* 12:19. 10.1186/s13072-019-0264-y PMC6427853 30898143

[B3] Altman J. Das G. D. (1965). Autoradiographic and histological evidence of postnatal hippocampal neurogenesis in rats. *J. Comp. Neurol.* 124 319–335. 10.1002/cne.901240303 5861717

[B4] Arlotta P. Molyneaux B. J. Chen J. Inoue J. Kominami R. Macklis J. D. (2005). Neuronal subtype-specific genes that control corticospinal motor neuron development in vivo. *Neuron* 45, 207–221 15664173 10.1016/j.neuron.2004.12.036

[B5] Arlotta P. Molyneaux B. J. Jabaudon D. Yoshida Y. Macklis J. D. (2008). Ctip2 controls the differentiation of medium spiny neurons and the establishment of the cellular architecture of the striatum. *J. Neurosci.* 28 622–632. 10.1523/jneurosci.2986-07.2008 18199763 PMC6670353

[B6] Avram D. Fields A. Pretty on Top K. Nevrivy D. J. Ishmael J. E. Leid M. (2000). Isolation of a novel family of C(2)H(2) zinc finger proteins implicated in transcriptional repression mediated by chicken ovalbumin upstream promoter transcription factor (COUP-TF) orphan nuclear receptors. *J. Biol. Chem.* 275 10315–10322. 10.1074/jbc.275.14.10315 10744719 PMC2819356

[B7] Avram D. Fields A. Senawong T. Topark-Ngarm A. Leid M. (2002). COUP-TF (chicken ovalbumin upstream promoter transcription factor)-interacting protein 1 (CTIP1) is a sequence-specific DNA binding protein. *Biochem. J.* 368 555–563. 10.1042/bj20020496 12196208 PMC1223006

[B8] Bae J. R. Kim S. H. (2017). Synapses in neurodegenerative diseases. *BMB Rep.* 50 237–246. 10.5483/bmbrep.2017.50.5.038 28270301 PMC5458673

[B9] Bagheri H. Badduke C. Qiao Y. Colnaghi R. Abramowicz I. Alcantara D. (2016). Identifying candidate genes for 2p15p16.1 microdeletion syndrome using clinical, genomic, and functional analysis. *JCI Insight* 1:e85461. 10.1172/jci.insight.85461 PMC5033885 27699255

[B10] Bagri A. Cheng H. J. Yaron A. Pleasure S. J. Tessier-Lavigne M. (2003). Stereotyped pruning of long hippocampal axon branches triggered by retraction inducers of the semaphorin family. *Cell* 113 285–299. 10.1016/s0092-8674(03)00267-8 12732138

[B11] Balci T. B. Sawyer S. L. Davila J. Humphreys P. Dyment D. A. (2015). Brain malformations in a patient with deletion 2p16.1: a refinement of the phenotype to BCL11A. *Eur. J. Med. Genet.* 58 351–354. 10.1016/j.ejmg.2015.04.006 25979662

[B12] Baranek C. Dittrich M. Parthasarathy S. Bonnon C. G. Britanova O. Lanshakov D. (2012). Protooncogene Ski cooperates with the chromatin-remodeling factor Satb2 in specifying callosal neurons. *Proc. Natl. Acad. Sci. U.S.A.* 109 3546–3551. 10.1073/pnas.1108718109 22334647 PMC3295291

[B13] Basak A. Hancarova M. Ulirsch J. C. Balci T. B. Trkova M. Pelisek M. (2015). BCL11A deletions result in fetal hemoglobin persistence and neurodevelopmental alterations. *J. Clin. Invest.* 125 2363–2368. 10.1172/jci81163 25938782 PMC4497765

[B14] Basak A. Munschauer M. Lareau C. A. Montbleau K. E. Ulirsch J. C. Hartigan C. R. (2020). Control of human hemoglobin switching by LIN28B-mediated regulation of BCL11A translation. *Nat. Genet.* 52, 138–145. 31959994 10.1038/s41588-019-0568-7 PMC7031047

[B15] Bauer D. E. Orkin S. H. (2015). Hemoglobin switching’s surprise: the versatile transcription factor BCL11A is a master repressor of fetal hemoglobin. *Curr. Opin. Genet. Dev.* 33 62–70. 10.1016/j.gde.2015.08.001 26375765 PMC4705561

[B16] Berg D. A. Su Y. Jimenez-Cyrus D. Patel A. Huang N. Morizet D. (2019). A common embryonic origin of stem cells drives developmental and adult neurogenesis. *Cell* 177 654.e15–668.e15. 30929900 10.1016/j.cell.2019.02.010 PMC6496946

[B17] Bogershausen N. Wollnik B. (2018). Mutational landscapes and phenotypic spectrum of SWI/SNF-related mental retardation disorders. *Front. Mol. Neurosci.* 11:252 10.3389/fnmol.2018.00252 PMC6085491 30123105

[B18] Boldrini M. Fulmore C. A. Tartt A. N. Simeon L. R. Pavlova I. Poposka V. (2018). Human hippocampal neurogenesis persists throughout aging. *Cell Stem Cell* 22 589.e5–599.e5. 29625071 10.1016/j.stem.2018.03.015 PMC5957089

[B19] Canovas J. Berndt F. A. Sepulveda H. Aguilar R. Veloso F. A. Montecino M. (2015). The specification of cortical subcerebral projection neurons depends on the direct repression of TBR1 by CTIP1/BCL11a. *J. Neurosci.* 35 7552–7564. 10.1523/jneurosci.0169-15.2015 25972180 PMC6705430

[B20] Chan C. M. Fulton J. Montiel-Duarte C. Collins H. M. Bharti N. Wadelin F. R. (2013). A signature motif mediating selective interactions of BCL11A with the NR2E/F subfamily of orphan nuclear receptors. *Nucleic Acids Res.* 41 9663–9679. 10.1093/nar/gkt761 23975195 PMC3834829

[B21] Chen B. Wang S. S. Hattox A. M. Rayburn H. Nelson S. B. Mcconnell S. K. (2008). The Fezf2-Ctip2 genetic pathway regulates the fate choice of subcortical projection neurons in the developing cerebral cortex. *Proc. Natl. Acad. Sci. U.S.A.* 105 11382–11387. 10.1073/pnas.0804918105 18678899 PMC2495013

[B22] Chesi A. Staahl B. T. Jovicic A. Couthouis J. Fasolino M. Raphael A. R. (2013). Exome sequencing to identify de novo mutations in sporadic ALS trios. *Nat. Neurosci.* 16 851–855. 10.1038/nn.3412 23708140 PMC3709464

[B23] Choi S. H. Bylykbashi E. Chatila Z. K. Lee S. W. Pulli B. Clemenson G. D. (2018). Combined adult neurogenesis and BDNF mimic exercise effects on cognition in an Alzheimer’s mouse model. *Science* 361:eaan8821. 10.1126/science.aan8821 30190379 PMC6149542

[B24] Cismasiu V. B. Adamo K. Gecewicz J. Duque J. Lin Q. Avram D. (2005). BCL11B functionally associates with the NuRD complex in T lymphocytes to repress targeted promoter. *Oncogene* 24 6753–6764. 10.1038/sj.onc.1208904 16091750

[B25] Cismasiu V. B. Duque J. Paskaleva E. Califano D. Ghanta S. Young H. A. (2009). BCL11B enhances TCR/CD28-triggered NF-kappaB activation through up-regulation of Cot kinase gene expression in T-lymphocytes. *Biochem. J.* 417 457–466. 10.1042/bj20080925 18831712 PMC2639648

[B26] Cismasiu V. B. Ghanta S. Duque J. Albu D. I. Chen H. M. Kasturi R. (2006). BCL11B participates in the activation of IL2 gene expression in CD4+ T lymphocytes. *Blood* 108 2695–2702. 10.1182/blood-2006-05-021790 16809611 PMC1895584

[B27] Cooper G. M. Coe B. P. Girirajan S. Rosenfeld J. A. Vu T. H. Baker C. (2011). A copy number variation morbidity map of developmental delay. *Nat. Genet.* 43 838–846. 10.1038/ng.909 21841781 PMC3171215

[B28] Dard R. F. Dahan L. Rampon C. (2018). Targeting hippocampal adult neurogenesis using transcription factors to reduce Alzheimer’s disease-associated memory impairments. *Hippocampus* 29 579–586. 10.1002/hipo.23052 30427560

[B29] De Bruyckere E. Simon R. Nestel S. Heimrich B. Katzel D. Egorov A. V. (2018). Stability and function of hippocampal mossy fiber synapses depend on Bcl11b/Ctip2. *Front. Mol. Neurosci.* 11:103 10.3389/fnmol.2018.00103 PMC5895709 29674952

[B30] De Rubeis S. He X. Goldberg A. P. Poultney C. S. Samocha K. Cicek A. E. (2014). Synaptic, transcriptional and chromatin genes disrupted in autism. *Nature* 515 209–215. 25363760 10.1038/nature13772 PMC4402723

[B31] Deciphering Developmental and Disorders Study (2015). Large-scale discovery of novel genetic causes of developmental disorders. *Nature* 519 223–228. 10.1038/nature14135 25533962 PMC5955210

[B32] Desplats P. A. Lambert J. R. Thomas E. A. (2008). Functional roles for the striatal-enriched transcription factor, Bcl11b, in the control of striatal gene expression and transcriptional dysregulation in Huntington’s disease. *Neurobiol. Dis.* 31 298–308. 10.1016/j.nbd.2008.05.005 18595722 PMC2569875

[B33] Dias C. Estruch S. B. Graham S. A. Mcrae J. Sawiak S. J. Hurst J. A. (2016). BCL11A haploinsufficiency causes an mental retardation and dysregulates transcription. *Am. J. Hum. Genet.* 99 253–274. 10.1016/j.ajhg.2016.05.030 27453576 PMC4974071

[B34] Dubuissez M. Loison I. Paget S. Vorng H. Ait-Yahia S. Rohr O. (2016). Protein kinase C-mediated phosphorylation of BCL11B at serine 2 negatively regulates its interaction with NuRD Complexes during CD4+ T-cell activation. *Mol. Cell. Biol.* 36 1881–1898. 10.1128/mcb.00062-16 27161321 PMC4911745

[B35] Fazel Darbandi S. Robinson Schwartz S. E. Qi Q. Catta-Preta R. Pai E. L. Mandell J. D. (2018). Neonatal Tbr1 dosage controls cortical layer 6 connectivity. *Neuron* 100:e837. 10.1016/j.neuron.2018.09.027 PMC6250594 30318412

[B36] Garthe A. Behr J. Kempermann G. (2009). Adult-generated hippocampal neurons allow the flexible use of spatially precise learning strategies. *PLoS One* 4:e5464. 10.1371/journal.pone.0005464 19421325 PMC2674212

[B37] Garthe A. Roeder I. Kempermann G. (2016). Mice in an enriched environment learn more flexibly because of adult hippocampal neurogenesis. *Hippocampus* 26 261–271. 10.1002/hipo.22520 26311488 PMC5049654

[B38] Golonzhka O. Leid M. Indra G. Indra A. K. (2007). Expression of COUP-TF-interacting protein 2 (CTIP2) in mouse skin during development and in adulthood. *Gene Expr. Patterns* 7 754–760. 10.1016/j.modgep.2007.06.002 17631058 PMC2063996

[B39] Grabarczyk P. Winkler P. Delin M. Sappa P. K. Bekeschus S. Hildebrandt P. (2018). The N-terminal CCHC zinc finger motif mediates homodimerization of transcription factor BCL11B. *Mol. Cell. Biol.* 38: e00368-17. 10.1128/MCB.00368-17 PMC5809685 29203643

[B40] Greig L. C. Woodworth M. B. Greppi C. Macklis J. D. (2016). Ctip1 controls acquisition of sensory area identity and establishment of sensory input fields in the developing neocortex. *Neuron* 90 261–277. 10.1016/j.neuron.2016.03.008 27100196 PMC4873772

[B41] Hancarova M. Simandlova M. Drabova J. Mannik K. Kurg A. Sedlacek Z. (2013). A patient with de novo 0.45 Mb deletion of 2p16.1: the role of BCL11A, PAPOLG, REL, and FLJ16341 in the 2p15-p16.1 microdeletion syndrome. *Am. J. Med. Genet. A* 161A 865–870. 10.1002/ajmg.a.35783 23495096

[B42] Harb K. Magrinelli E. Nicolas C. S. Lukianets N. Frangeul L. Pietri M. (2016). Area-specific development of distinct projection neuron subclasses is regulated by postnatal epigenetic modifications. *eLife* 5:e09531. 10.7554/eLife.09531 PMC4744182 26814051

[B43] Henstridge C. M. Tzioras M. Paolicelli R. C. (2019). Glial contribution to excitatory and inhibitory synapse loss in neurodegeneration. *Front. Cell Neurosci.* 13:63. 10.3389/fncel.2019.00063 30863284 PMC6399113

[B44] Hevner R. F. Shi L. Justice N. Hsueh Y. Sheng M. Smiga S. (2001). Tbr1 regulates differentiation of the preplate and layer 6. *Neuron* 29 353–366. 10.1016/s0896-6273(01)00211-2 11239428

[B45] Hong S. Beja-Glasser V. F. Nfonoyim B. M. Frouin A. Li S. Ramakrishnan S. (2016). Complement and microglia mediate early synapse loss in Alzheimer mouse models. *Science* 352 712–716. 10.1126/science.aad8373 27033548 PMC5094372

[B46] Hsieh J. (2012). Orchestrating transcriptional control of adult neurogenesis. *Genes Dev.* 26 1010–1021. 10.1101/gad.187336.112 22588716 PMC3360557

[B47] Iossifov I. Ronemus M. Levy D. Wang Z. Hakker I. Rosenbaum J. (2012). De novo gene disruptions in children on the autistic spectrum. *Neuron* 74 285–299. 22542183 10.1016/j.neuron.2012.04.009 PMC3619976

[B48] Jain S. Lafrancois J. J. Botterill J. J. Alcantara-Gonzalez D. Scharfman H. E. (2019). Adult neurogenesis in the mouse dentate gyrus protects the hippocampus from neuronal injury following severe seizures. *Hippocampus* 29 683–709. 10.1002/hipo.23062 30672046 PMC6640126

[B49] John A. Brylka H. Wiegreffe C. Simon R. Liu P. Juttner R. (2012). Bcl11a is required for neuronal morphogenesis and sensory circuit formation in dorsal spinal cord development. *Development* 139 1831–1841. 10.1242/dev.072850 22491945 PMC4067532

[B50] Kadoch C. Crabtree G. R. (2015). Mammalian SWI/SNF chromatin remodeling complexes and cancer: mechanistic insights gained from human genomics. *Sci. Adv.* 1:e1500447. 10.1126/sciadv.1500447 26601204 PMC4640607

[B51] Kadoch C. Hargreaves D. C. Hodges C. Elias L. Ho L. Ranish J. (2013). Proteomic and bioinformatic analysis of mammalian SWI/SNF complexes identifies extensive roles in human malignancy. *Nat. Genet.* 45 592–601. 10.1038/ng.2628 23644491 PMC3667980

[B52] Kempermann G. Gage F. H. Aigner L. Song H. Curtis M. A. Thuret S. (2018). Human adult neurogenesis: evidence and remaining questions. *Cell Stem Cell* 23 25–30. 10.1016/j.stem.2018.04.004 29681514 PMC6035081

[B53] Kuipers S. D. Schroeder J. E. Trentani A. (2015). Changes in hippocampal neurogenesis throughout early development. *Neurobiol. Aging* 36 365–379. 10.1016/j.neurobiolaging.2014.07.033 25172123

[B54] Kumar R. Wang R. A. (2016). Structure, expression and functions of MTA genes. *Gene* 582 112–121. 10.1016/j.gene.2016.02.012 26869315 PMC4785049

[B55] Kuo T. Y. Chen C. Y. Hsueh Y. P. (2010a). Bcl11A/CTIP1 mediates the effect of the glutamate receptor on axon branching and dendrite outgrowth. *J. Neurochem.* 114 1381–1392. 20534004 10.1111/j.1471-4159.2010.06852.x

[B56] Kuo T. Y. Hong C. J. Chien H. L. Hsueh Y. P. (2010b). X-linked mental retardation gene CASK interacts with Bcl11A/CTIP1 and regulates axon branching and outgrowth. *J. Neurosci. Res.* 88 2364–2373. 20623620 10.1002/jnr.22407

[B57] Kuo T. Y. Hong C. J. Hsueh Y. P. (2009). Bcl11A/CTIP1 regulates expression of DCC and MAP1b in control of axon branching and dendrite outgrowth. *Mol. Cell. Neurosci.* 42 195–207. 10.1016/j.mcn.2009.07.006 19616629

[B58] Lai A. Y. Wade P. A. (2011). Cancer biology and NuRD: a multifaceted chromatin remodelling complex. *Nat. Rev. Cancer* 11 588–596. 10.1038/nrc3091 21734722 PMC4157524

[B59] Lee E. Chung W. S. (2019). Glial control of synapse number in healthy and diseased brain. *Front. Cell Neurosci.* 13:42. 10.3389/fncel.2019.00042 30814931 PMC6381066

[B60] Leid M. Ishmael J. E. Avram D. Shepherd D. Fraulob V. Dolle P. (2004). CTIP1 and CTIP2 are differentially expressed during mouse embryogenesis. *Gene Expr. Patterns* 4 733–739. 10.1016/j.modgep.2004.03.009 15465497 PMC2819357

[B61] Lennon M. J. Jones S. P. Lovelace M. D. Guillemin G. J. Brew B. J. (2016). Bcl11b: a new piece to the complex puzzle of amyotrophic lateral sclerosis neuropathogenesis? *Neurotox. Res.* 29 201–207. 10.1007/s12640-015-9573-5 26563995

[B62] Leone D. P. Heavner W. E. Ferenczi E. A. Dobreva G. Huguenard J. R. Grosschedl R. (2015). Satb2 regulates the differentiation of both callosal and subcerebral projection neurons in the developing cerebral cortex. *Cereb. Cortex* 25 3406–3419. 10.1093/cercor/bhu156 25037921 PMC4585495

[B63] Lessel D. Gehbauer C. Bramswig N. C. Schluth-Bolard C. Venkataramanappa S. Van Gassen K. L. I. (2018). BCL11B mutations in patients affected by a neurodevelopmental disorder with reduced type 2 innate lymphoid cells. *Brain* 141 2299–2311. 10.1093/brain/awy173 29985992 PMC6061686

[B64] Liu H. Ippolito G. C. Wall J. K. Niu T. Probst L. Lee B. S. (2006). Functional studies of BCL11A: characterization of the conserved BCL11A-XL splice variant and its interaction with BCL6 in nuclear paraspeckles of germinal center B cells. *Mol. Cancer* 5:18. 10.1186/1476-4598-5-18 PMC1526750 16704730

[B65] Liu N. Hargreaves V. V. Zhu Q. Kurland J. V. Hong J. Kim W. (2018). Direct promoter repression by BCL11A controls the fetal to adult hemoglobin switch. *Cell* 173 430.e17–442.e17. 29606353 10.1016/j.cell.2018.03.016 PMC5889339

[B66] Liu X. D. Zhu X. N. Halford M. M. Xu T. L. Henkemeyer M. Xu N. J. (2018). Retrograde regulation of mossy fiber axon targeting and terminal maturation via postsynaptic Lnx1. *J. Cell Biol.* 217 4007–4024. 10.1083/jcb.201803105 30185604 PMC6219728

[B67] Llorens-Martin M. (2018). Exercising new neurons to vanquish alzheimer disease. *Brain Plast.* 4 111–126. 10.3233/bpl-180065 30564550 PMC6296267

[B68] Matsuda K. (2017). Synapse organization and modulation via C1q family proteins and their receptors in the central nervous system. *Neurosci. Res.* 116 46–53. 10.1016/j.neures.2016.11.004 27845167

[B69] Matsuda K. Budisantoso T. Mitakidis N. Sugaya Y. Miura E. Kakegawa W. (2016). Transsynaptic modulation of kainate receptor functions by C1q-like proteins. *Neuron* 90 752–767. 10.1016/j.neuron.2016.04.001 27133466

[B70] McKenna W. L. Ortiz-Londono C. F. Mathew T. K. Hoang K. Katzman S. Chen B. (2015). Mutual regulation between Satb2 and Fezf2 promotes subcerebral projection neuron identity in the developing cerebral cortex. *Proc. Natl. Acad. Sci. U.S.A.* 112 11702–11707. 10.1073/pnas.1504144112 26324926 PMC4577201

[B71] Moreno-Jimenez E. P. Flor-Garcia M. Terreros-Roncal J. Rabano A. Cafini F. Pallas-Bazarra N. (2019). Adult hippocampal neurogenesis is abundant in neurologically healthy subjects and drops sharply in patients with Alzheimer’s disease. *Nat. Med.* 25 554–560. 10.1038/s41591-019-0375-9 30911133

[B72] Nakamura T. Yamazaki Y. Saiki Y. Moriyama M. Largaespada D. A. Jenkins N. A. (2000). Evi9 encodes a novel zinc finger protein that physically interacts with BCL6, a known human B-cell proto-oncogene product. *Mol. Cell. Biol.* 20 3178–3186. 10.1128/mcb.20.9.3178-3186.2000 10757802 PMC85612

[B73] Noctor S. C. Martinez-Cerdeno V. Ivic L. Kriegstein A. R. (2004). Cortical neurons arise in symmetric and asymmetric division zones and migrate through specific phases. *Nat. Neurosci.* 7 136–144. 10.1038/nn1172 14703572

[B74] Nord A. S. Pattabiraman K. Visel A. Rubenstein J. L. R. (2015). Genomic perspectives of transcriptional regulation in forebrain development. *Neuron* 85 27–47. 10.1016/j.neuron.2014.11.011 25569346 PMC4438709

[B75] O’Connor T. P. Cockburn K. Wang W. Tapia L. Currie E. Bamji S. X. (2009). Semaphorin 5B mediates synapse elimination in hippocampal neurons. *Neural Dev.* 4:18. 10.1186/1749-8104-4-18 19463192 PMC2696441

[B76] Ohara S. Onodera M. Simonsen O. W. Yoshino R. Hioki H. Iijima T. (2018). Intrinsic projections of layer Vb neurons to layers Va, III, and II in the lateral and medial entorhinal cortex of the rat. *Cell Rep.* 24 107–116. 10.1016/j.celrep.2018.06.014 29972772

[B77] Paolicelli R. C. Bolasco G. Pagani F. Maggi L. Scianni M. Panzanelli P. (2011). Synaptic pruning by microglia is necessary for normal brain development. *Science* 333 1456–1458. 10.1126/science.1202529 21778362

[B78] Peron A. Bradbury K. Viskochil D. H. Dias C. (2019). “BCL11A-related mental retardation,” in *GeneReviews((R))*, eds Adam M. P. Ardinger H. H. Pagon R. A. Wallace S. E. Bean L. J. H. Stephens K. (Seattle: University of Washington), 1993–2020. 31556984

[B79] Peter B. Matsushita M. Oda K. Raskind W. (2014). De novo microdeletion of BCL11A is associated with severe speech sound disorder. *Am. J. Med. Genet. A* 164A 2091–2096. 10.1002/ajmg.a.36599 24810580

[B80] Punwani D. Zhang Y. Yu J. Cowan M. J. Rana S. Kwan A. (2016). Multisystem anomalies in severe combined immunodeficiency with mutant BCL11B. *N. Engl. J. Med.* 375 2165–2176. 10.1056/nejmoa1509164 27959755 PMC5215776

[B81] Rajcan-Separovic E. Harvard C. Liu X. Mcgillivray B. Hall J. G. Qiao Y. (2007). Clinical and molecular cytogenetic characterisation of a newly recognised microdeletion syndrome involving 2p15-16.1. *J. Med. Genet.* 44 269–276. 10.1136/jmg.2006.045013 16963482 PMC2598046

[B82] Riccomagno M. M. Hurtado A. Wang H. Macopson J. G. Griner E. M. Betz A. (2012). The RacGAP beta2-Chimaerin selectively mediates axonal pruning in the hippocampus. *Cell* 149 1594–1606. 10.1016/j.cell.2012.05.018 22726444 PMC3395473

[B83] Ronan J. L. Wu W. Crabtree G. R. (2013). From neural development to cognition: unexpected roles for chromatin. *Nat. Rev. Genet.* 14 347–359. 10.1038/nrg3413 23568486 PMC4010428

[B84] Sanders S. J. He X. Willsey A. J. Ercan-Sencicek A. G. Samocha K. E. Cicek A. E. (2015). Insights into autism spectrum disorder genomic architecture and biology from 71 risk loci. *Neuron* 87 1215–1233. 26402605 10.1016/j.neuron.2015.09.016 PMC4624267

[B85] Satterwhite E. Sonoki T. Willis T. G. Harder L. Nowak R. Arriola E. L. (2001). The BCL11 gene family: involvement of BCL11A in lymphoid malignancies. *Blood* 98 3413–3420. 10.1182/blood.v98.12.3413 11719382

[B86] Schizophrenia Working Group of the Psychiatric Genomics Consortium (2014). Biological insights from 108 schizophrenia-associated genetic loci. *Nature* 511 421–427. 10.1038/nature13595 25056061 PMC4112379

[B87] Sellgren C. M. Gracias J. Watmuff B. Biag J. D. Thanos J. M. Whittredge P. B. (2019). Increased synapse elimination by microglia in schizophrenia patient-derived models of synaptic pruning. *Nat. Neurosci.* 22 374–385. 10.1038/s41593-018-0334-7 30718903 PMC6410571

[B88] Senawong T. Peterson V. J. Avram D. Shepherd D. M. Frye R. A. Minucci S. (2003). Involvement of the histone deacetylase SIRT1 in chicken ovalbumin upstream promoter transcription factor (COUP-TF)-interacting protein 2-mediated transcriptional repression. *J. Biol. Chem.* 278 43041–43050. 10.1074/jbc.m307477200 12930829 PMC2819354

[B89] Senawong T. Peterson V. J. Leid M. (2005). BCL11A-dependent recruitment of SIRT1 to a promoter template in mammalian cells results in histone deacetylation and transcriptional repression. *Arch. Biochem. Biophys.* 434 316–325. 10.1016/j.abb.2004.10.028 15639232 PMC2819353

[B90] Simon R. Baumann L. Fischer J. Seigfried F. A. De Bruyckere E. Liu P. (2016). Structure-function integrity of the adult hippocampus depends on the transcription factor Bcl11b/Ctip2. *Genes Brain Behav.* 15 405–419. 10.1111/gbb.12287 26915960 PMC4832350

[B91] Simon R. Brylka H. Schwegler H. Venkataramanappa S. Andratschke J. Wiegreffe C. (2012). A dual function of Bcl11b/Ctip2 in hippocampal neurogenesis. *EMBO J.* 31 2922–2936. 10.1038/emboj.2012.142 22588081 PMC3395096

[B92] Snyder J. S. (2019). Recalibrating the relevance of adult neurogenesis. *Trends Neurosci.* 42 164–178. 10.1016/j.tins.2018.12.001 30686490

[B93] Soblet J. Dimov I. Graf Von Kalckreuth C. Cano-Chervel J. Baijot S. Pelc K. (2018). BCL11A frameshift mutation associated with dyspraxia and hypotonia affecting the fine, gross, oral, and speech motor systems. *Am. J. Med. Genet. A* 176 201–208. 10.1002/ajmg.a.38479 28960836 PMC5765401

[B94] Sokpor G. Xie Y. Rosenbusch J. Tuoc T. (2017). Chromatin remodeling BAF (SWI/SNF) complexes in neural development and disorders. *Front. Mol. Neurosci.* 10:243 10.3389/fnmol.2017.00243 PMC5540894 28824374

[B95] Son E. Y. Crabtree G. R. (2014). The role of BAF (mSWI/SNF) complexes in mammalian neural development. *Am. J. Med. Genet. C Semin. Med. Genet.* 166C 333–349. 10.1002/ajmg.c.31416 25195934 PMC4405377

[B96] Sorrells S. F. Paredes M. F. Cebrian-Silla A. Sandoval K. Qi D. Kelley K. W. (2018). Human hippocampal neurogenesis drops sharply in children to undetectable levels in adults. *Nature* 555 377–381. 10.1038/nature25975 29513649 PMC6179355

[B97] Spalding K. L. Bergmann O. Alkass K. Bernard S. Salehpour M. Huttner H. B. (2013). Dynamics of hippocampal neurogenesis in adult humans. *Cell* 153 1219–1227. 23746839 10.1016/j.cell.2013.05.002 PMC4394608

[B98] Stephan A. H. Barres B. A. Stevens B. (2012). The complement system: an unexpected role in synaptic pruning during development and disease. *Annu. Rev. Neurosci.* 35 369–389. 10.1146/annurev-neuro-061010-113810 22715882

[B99] Surmeli G. Marcu D. C. Mcclure C. Garden D. L. F. Pastoll H. Nolan M. F. (2015). Molecularly defined circuitry reveals input-output segregation in deep layers of the medial entorhinal cortex. *Neuron* 88 1040–1053. 10.1016/j.neuron.2015.10.041 26606996 PMC4675718

[B100] Topark-Ngarm A. Golonzhka O. Peterson V. J. Barrett B. Jr. Martinez B. (2006). CTIP2 associates with the NuRD complex on the promoter of p57KIP2, a newly identified CTIP2 target gene. *J. Biol. Chem.* 281 32272–32283. 10.1074/jbc.m602776200 16950772 PMC2547407

[B101] Tuoc T. Dere E. Radyushkin K. Pham L. Nguyen H. Tonchev A. B. (2017). Ablation of BAF170 in developing and postnatal dentate gyrus affects neural stem cell proliferation, differentiation, and learning. *Mol. Neurobiol.* 54 4618–4635. 10.1007/s12035-016-9948-5 27392482 PMC5509785

[B102] Urban N. Guillemot F. (2014). Neurogenesis in the embryonic and adult brain: same regulators, different roles. *Front. Cell. Neurosci.* 8:396. 10.3389/fncel.2014.00396 25505873 PMC4245909

[B103] Vivar C. Potter M. C. Van Praag H. (2013). All about running: synaptic plasticity, growth factors and adult hippocampal neurogenesis. *Curr. Top. Behav. Neurosci.* 15 189–210. 10.1007/7854_2012_220 22847651 PMC4565722

[B104] Vogel Ciernia A. Kramar E. A. Matheos D. P. Havekes R. Hemstedt T. J. Magnan C. N. (2017). Mutation of neuron-specific chromatin remodeling subunit BAF53b: rescue of plasticity and memory by manipulating actin remodeling. *Learn. Mem.* 24 199–209. 10.1101/lm.044602.116 28416631 PMC5397687

[B105] Vogel-Ciernia A. Matheos D. P. Barrett R. M. Kramar E. A. Azzawi S. Chen Y. (2013). The neuron-specific chromatin regulatory subunit BAF53b is necessary for synaptic plasticity and memory. *Nat. Neurosci.* 16 552–561. 10.1038/nn.3359 23525042 PMC3777648

[B106] Vogel-Ciernia A. Wood M. A. (2014). Examining object location and object recognition memory in mice. *Curr. Protoc. Neurosci.* 69 31–17. 25297693 10.1002/0471142301.ns0831s69 PMC4219523

[B107] Whitton L. Apostolova G. Rieder D. Dechant G. Rea S. Donohoe G. (2018). Genes regulated by SATB2 during neurodevelopment contribute to schizophrenia and educational attainment. *PLoS Genet.* 14:e1007515. 10.1371/journal.pgen.1007515 30040823 PMC6097700

[B108] Whitton L. Cosgrove D. Clarkson C. Harold D. Kendall K. Richards A. (2016). Cognitive analysis of schizophrenia risk genes that function as epigenetic regulators of gene expression. *Am. J. Med. Genet. B Neuropsychiatr. Genet.* 171 1170–1179. 10.1002/ajmg.b.32503 27762073

[B109] Wiegreffe C. Simon R. Peschkes K. Kling C. Strehle M. Cheng J. (2015). Bcl11a (Ctip1) controls migration of cortical projection neurons through regulation of sema3c. *Neuron* 87 311–325. 10.1016/j.neuron.2015.06.023 26182416

[B110] Wilton D. K. Dissing-Olesen L. Stevens B. (2019). Neuron-glia signaling in synapse elimination. *Annu. Rev. Neurosci.* 42 107–127. 10.1146/annurev-neuro-070918-050306 31283900

[B111] Woodworth M. B. Greig L. C. Liu K. X. Ippolito G. C. Tucker H. O. Macklis J. D. (2016). Ctip1 regulates the balance between specification of distinct projection neuron subtypes in deep cortical layers. *Cell Rep.* 15 999–1012. 10.1016/j.celrep.2016.03.064 27117402 PMC4873759

[B112] Xu N. J. Henkemeyer M. (2009). Ephrin-B3 reverse signaling through Grb4 and cytoskeletal regulators mediates axon pruning. *Nat. Neurosci.* 12 268–276. 10.1038/nn.2254 19182796 PMC2661084

[B113] Yoo M. Choi K. Y. Kim J. Kim M. Shim J. Choi J. H. (2017). BAF53b, a neuron-specific nucleosome remodeling factor, is induced after learning and facilitates long-term memory consolidation. *J. Neurosci.* 37 3686–3697. 10.1523/jneurosci.3220-16.2017 28270570 PMC6596926

[B114] Yoshida M. Nakashima M. Okanishi T. Kanai S. Fujimoto A. Itomi K. (2018). Identification of novel BCL11A variants in patients with epileptic encephalopathy: expanding the phenotypic spectrum. *Clin. Genet.* 93 368–373. 10.1111/cge.13067 28589569

[B115] Yuzaki M. (2017). The C1q complement family of synaptic organizers: not just complementary. *Curr. Opin. Neurobiol.* 45 9–15. 10.1016/j.conb.2017.02.002 28219683

[B116] Yuzaki M. (2018). Two classes of secreted synaptic organizers in the central nervous system. *Annu. Rev. Physiol.* 80 243–262. 10.1146/annurev-physiol-021317-121322 29166241

[B117] Zhang L. J. Bhattacharya S. Leid M. Ganguli-Indra G. Indra A. K. (2012). Ctip2 is a dynamic regulator of epidermal proliferation and differentiation by integrating EGFR and notch signaling. *J. Cell Sci.* 125 5733–5744. 10.1242/jcs.108969 23015591 PMC3575708

